# A Novel Artificial Bee Colony Algorithm Based on Internal-Feedback Strategy for Image Template Matching

**DOI:** 10.1155/2014/906861

**Published:** 2014-04-29

**Authors:** Bai Li, Li-Gang Gong, Ya Li

**Affiliations:** ^1^School of Control Science and Engineering, Zhejiang University, Hangzhou 310027, China; ^2^School of Automation Science and Electrical Engineering, Beihang University, Beijing 100191, China; ^3^School of Mathematics and Systems Science & LMIB, Beihang University, Beijing 100191, China

## Abstract

Image template matching refers to the technique of locating a given reference image over a source image such that they are the most similar. It is a fundamental mission in the field of visual target recognition. In general, there are two critical aspects of a template matching scheme. One is similarity measurement and the other is best-match location search. In this work, we choose the well-known normalized cross correlation model as a similarity criterion. The searching procedure for the best-match location is carried out through an internal-feedback artificial bee colony (IF-ABC) algorithm. IF-ABC algorithm is highlighted by its effort to fight against premature convergence. This purpose is achieved through discarding the conventional roulette selection procedure in the ABC algorithm so as to provide each employed bee an equal chance to be followed by the onlooker bees in the local search phase. Besides that, we also suggest efficiently utilizing the internal convergence states as feedback guidance for searching intensity in the subsequent cycles of iteration. We have investigated four ideal template matching cases as well as four actual cases using different searching algorithms. Our simulation results show that the IF-ABC algorithm is more effective and robust for this template matching mission than the conventional ABC and two state-of-the-art modified ABC algorithms do.

## 1. Introduction

Template matching is defined as the action of recognizing predefined template patterns in a source image. It is a fundamental issue in pattern recognition and has been widely applied to the fields such as face recognition [1], pulmonary nodules detection [2], handwriting identification [3], and road detection [4] over the past few decades.

In general, template matching involves two critical aspects: similarity measurement and best-match search [5, 6]. When measuring the similarity, a source image and a predefined template image are superimposed in a certain location and then the similarity evaluation is made on the basis of a selected model or criterion. The sum of absolute differences (SAD), the sum of squared differences (SSD), and the normalized cross correlation (NCC) are all popular similarity measurement models. SAD is sensitive to the illumination disturbances and may lead to large variations in the intensity values [7]. SSD has a similar drawback. The NCC model utilizes rotation and scale invariant evaluations for the degree of similarity [8] and is confirmed to be more robust than SAD and SSD, especially in terms of uniform illumination changes in the source image [6, 9]. Therefore, the NCC model is more widely used than the other two models. With regard to the search strategy for a best-match position, a thorough search algorithm is proposed as a pioneering work [10], in which all the pixel-candidate positions are checked until the one with the maximum similarity is located. However, such exhaustive search is computationally expensive, which restricts its applications, especially in terms of some real-time recognition issues.

In order to reduce the computation complexity in the template matching schemes, search strategies based on evolutionary algorithms have been developed and investigated. Species-based genetic algorithm (Sb-GA) [11], bat algorithm (BA) [12], chaotic quantum-behaved particle swarm optimization (CQ-PSO) [13], chaotic imperialist competitive algorithm (C-ICA) [14], and a states-of-matter search (SMS) algorithm [5] have been proposed for this template matching problem. Although these algorithms aim to reduce the computation load of the global optimums searching, they cannot avoid the derivation of suboptimal matching results. It is worth pointing out that most evolutionary algorithms are generally suitable for the optimization of convex or nearly convex functions [15]. But the template matching field contains a finite number of candidate matching locations. Such a discrete function is usually anything but smooth. In other words, the objective function concerning template matching may drastically oscillate along the domain, which critically restricts the advantages of such evolutionary algorithms. Therefore, it calls for a new way to modify these existing evolutionary algorithms so as to accommodate the discreteness and oscillation in an objective function.

Artificial bee colony (ABC) is a relatively new swarm intelligence algorithm. It is motivated by the foraging behavior of bee swarms, in which both local exploitation and global exploration are implemented [16]. Applications and developments of this algorithm have been proposed in a variety of ways [17–38]. In this paper, our previously proposed internal-feedback artificial bee colony (IF-ABC) algorithm is adopted as a search approach to find the best-match location for the template matching scheme. IF-ABC features by its effort to fight against premature convergence. In a sense, IF-ABC sacrifices part of its convergence speed for the ability to avoid premature convergence [22, 39, 40]. Our work intends to have an intensive evaluation about the true performance of IF-ABC regarding image template matching, in comparison with some other state-of-the-art ABCs.

The remainder of this paper is organized as follows. In Sections 2 and 3, basic principles of NCC model and the conventional ABC algorithm are introduced.  Section 4 holds a description of IF-ABC algorithm in detail. Then, several cases of comparative experiments have been conducted in Section 5. Further discussions concerning the comparable convergence performances are included in Section 6. The final section includes the conclusions, the limitations of this study, and our future work.

## 2. Model of Image Template Matching

**Figure 1:**
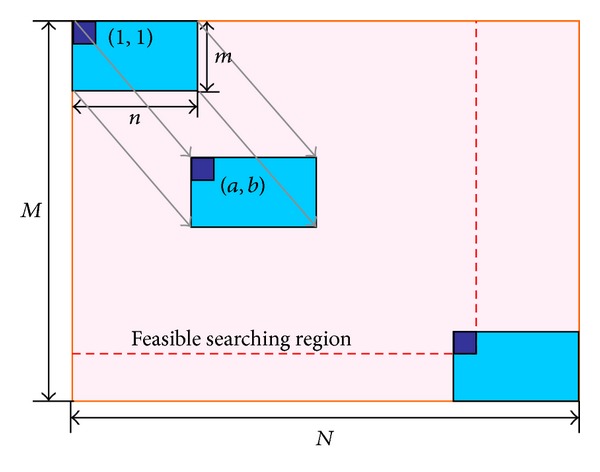
Schematic diagram of template matching process.

Image template matching aims to locate a given reference image (which is also called template image) over a source image such that they best match each other. Typically it is assumed that the predefined template image and the source image are given in RGB format. Before the matching process starts, the corresponding grayscale images are derived in the preprocessing procedure. The grayscale template image is described by a matrix temp_*m*×*n*_, in which temp(*a*, *b*) refers to the gray level of the pixel located at (*a*, *b*) in the grayscale template image. It is obvious that temp(*a*, *b*)∈[0,255]∩*Z*. Similarly, the grayscale source image is described by a matrix test_*M*×*N*_. The template matching scheme can be expressed as finding an optimal location (*a*, *b*) for the grayscale template image temp_*m*×*n*_ so that the similarity between temp(1 : *m*, 1 : *n*) and test(*a* : (*a* + *m* − 1), *b* : (*b* + *n* − 1)) is maximized within the feasible searching region (see Figure 1).

There are a number of ways to measure such similarity. Considering the robustness in terms of disturbing illuminations, we choose the following NCC model (see (1)) in this work. Consider

(1) NCC(a,b)≜(∑x=1m∑y=1n[temp(a+x−1,b+y−1)·test(x,y)])  ×(∑x=1m∑y=1n[temp2(a+x−1,b+y−1)]      ·∑x=1m∑y=1n[test2(x,y)])−1,

where (*a*, *b*) represents the target location of top-left-corner pixel in the grayscale template. In other words, the grayscale template image shifts according to a vector of **β** = (*a*, *b*) over the grayscale source image. In this particular case, it is required that *a* ∈ [1, *M* − *m* + 1]∩*Z*,*b* ∈ [1, *N* − *n* + 1]∩*Z*, which are regarded as the feasible searching conditions as demonstrated in Figure 1. It is easy to see that NCC(·)∈[0,1] and the optimal (a*, b*) satisfies NCC(a*, b*) = 1.

Implementation procedures of template matching are given as follows.

**Step 1**

Import original source image and predefined template image (both in RGB form).

**Step 2**

Convert template and source image to grayscale.

**Step 3**

Choose NCC model as similarity criteria.

**Step 4**

Search among all feasible locations aiming to find the best-match location using IF-ABC algorithm.

**Step 5**

Terminate searching procedure when terminal condition is reached; then output best-match result.

## 3. Brief Review of Conventional ABC Algorithm

The preceding section introduces the NCC model, which is taken as a similarity criterion in this work. But how can one find the very (a*, b*) that maximizes NCC? In this section and the next, two intelligent algorithms named ABC and IF-ABC will be introduced, respectively, either of which can be taken as a searching approach for the optimal matching location.

ABC is a swarm intelligence-based optimization algorithm. It is inspired by the forging behavior of bees. In this algorithm, there are three kinds of bees, namely, the employed bees, the onlooker bees, and the scout bees. They cooperate to search for the optimal nectar source in the space [16, 23].

At the beginning, an initial population is randomly generated, which contains as many as *SN* food sources (i.e., SN feasible solutions), using (2). Consider

(2) Xi⟵Xmin⁡+rand(0,1)·(Xmax⁡−Xmin⁡),i=1,2,…,SN. 

In the above equation, each solution **X**_*i*_ = (*x*_*i*_^1^, *x*_*i*_^2^,…, *x*_*i*_^*D*^) is a *D*-dimensional vector, **X**_max⁡_ and **X**_min⁡_ are the predefined constraints set for the optimization problem, and rand(0,1) denotes a random number in the range (0,1) obeying the uniform distribution.

Then, the iteration process starts. Generally, as many as SN employed bees search globally in each cycle of iteration, and then SN onlooker bees search locally around the “qualified” employed bees. Those employed bees who cannot make any progress within some certain cycles will be replaced by the scout bees. The qualification standard concerns the roulette selection strategy and will be introduced later.

In detail, each employed bee utilizes the position of its randomly chosen companion to generate a new searching direction, as shown in (3). Consider

(3) xij⟵xij+rand(−1,1)·(xkj−xij),j≠k, j,k∈[1,D]∩Z, i∈[1,SN]∩Z.

Here, **X**_*i*_ = (*x*_*i*_^1^, *x*_*i*_^2^,…, *x*_*i*_^*D*^) denotes the position of the *i*th employed bee, **X**_*k*_ = (*x*_*k*_^1^, *x*_*k*_^2^,…, *x*_*k*_^*D*^) stands for the position of a randomly chosen companion, and the searching location changes in the *j*th element of **X**_*i*_.

Thereafter, the greedy selection procedure is implemented. If the new position updated by (3) is better (i.e., the corresponding objective function value is higher), the previous position is discarded; otherwise, the employed bee remains at the previous position. When all the SN employed bees complete the searching procedure mentioned above, an index *P* is calculated as the qualification measurement for the employed bees using (4). Consider

(4) $$\begin{array}{c} P\left(i\right)=\frac{\text{fitness}\left(i\right)}{\sum_{j=1}^{\text{SN}}\text{fitness}\left(j\right)},\\ \text{fitness}\left(i\right)=\left\{\begin{array}{c} \frac{1}{1+\text{obj}\left(X_{i}\right)}\;\;\text{if}\;\;\text{obj}\left(X_{i}\right)\geq 0\\ 1+\text{abs}\left(\text{obj}\left(X_{i}\right)\right)\;\;\text{if}\;\;\text{obj}\left(X_{i}\right)<0 \end{array}\right\}, \end{array}$$

where obj(·) denotes the objective function, and fitness(·) is conventionally defined. Each onlooker bee needs to search around an employed bee using (3). In this case, *x*_*k*_^*j*^ stands for the corresponding element of the selected employed bee, and *x*_*i*_^*j*^ denotes that of the *i*th onlooker bee. Again, the greedy selection procedure is implemented here.

The selection principle for the qualified employed bees concerns the roulette selection strategy. If *P*_1_ ≥ rand(0,1), the first employed bee is chosen for the specific onlooker bee; otherwise, comparison between *P*_2_ and rand(0,1) is carried on. If all the *P*_*i*_ are smaller than rand(0,1), such process goes over again until one employed bee satisfies the condition. In this way, each of the SN onlooker bees determines which employed bee to follow, respectively.

During each cycle of the iteration, once the *i*th employed bee or an onlooker bee (which searches around the *i*th employed bee) finds a better position in the crossover procedure, the parameter trial(*i*) is directly reset to zero; otherwise, it is added by one. In this sense, trial is regarded as a counter recording the invalid searching times around the *i*th employed bee. Before a new cycle of iteration starts, it is necessary to check whether any trial(*i*) exceeds a certain threshold Limit. If trail(*i*) > Limit, the *i*th employed bee will be directly replaced by a scout bee. A scout bee simply stands for a randomly initialized position utilizing (2).

## 4. Principle of IF-ABC Algorithm

IF-ABC algorithm was originally proposed to optimize protein secondary structures in our previous works [22, 40]. In this paper, it is slightly modified to accommodate well to the template matching problem.

At first, as many as SN employed bees are randomly sent out to explore in the feasible solution space using (2). Then, the iteration process gets started. In each cycle of iteration, an employed bee utilizes the location of one randomly selected companion to generate a new searching location using (3). Afterwards, the onlooker bees carry on the searching process.

In the IF-ABC algorithm, each of the employed bees is given a chance to be followed by an onlooker no matter whether they are “qualified” or not. In other words, we discard the conventional roulette selection procedure mentioned in ABC here, pursuing to bring more chances (i.e., more dynamics and indeterminacy) to the evolution process. Therefore, the *i*th onlooker bee in IF-ABC directly chooses the *i*th employed bees to follow. At this point, a new idea is proposed (see (5)), which presents a new principle for onlooker bees to follow the employed bees. It is notable that we introduce a multiplier *γ*(*i*) in this equation, which is defined in (6). Consider

(5) xij⟵xij+γ(i)·rand(−1,1)·(xkj−xij),j≠k, j,k∈[1,D]∩Z, i∈[1,SN]∩Z,

(6) $$\begin{array}{c} \gamma \left(i\right)=\exp\left(\left(\text{trial}\left(i\right)-1\right)\cdot \frac{\ln\alpha }{\text{Limit}-1}\right). \end{array}$$

Similar to ABC, the parameter trial in (6) records the number of inefficient searching times. In IF-ABC, if the *i*th employed/onlooker bee finds a position that is better than the previous one it stays at, trial(*i*) is reset to 1 (but not zero); otherwise, we add 1 to it. If trial(*i*) exceeds Limit, the *i*th employed bee will be reinitialized using (2). *γ*(*i*) manipulates the exploitation accuracy, which decreases exponentially to *α* as trial(*i*) gradually approaches Limit. Here, *α* is a user-specified lower boundary of convergent scale. In this sense, the search process is intensified gradually with an increasing trial(*i*). That is, the search accuracy will be gradually enhanced before the position of the *i*th employed bee is eventually discarded by the whole bee swarm.

**Figure 2:**
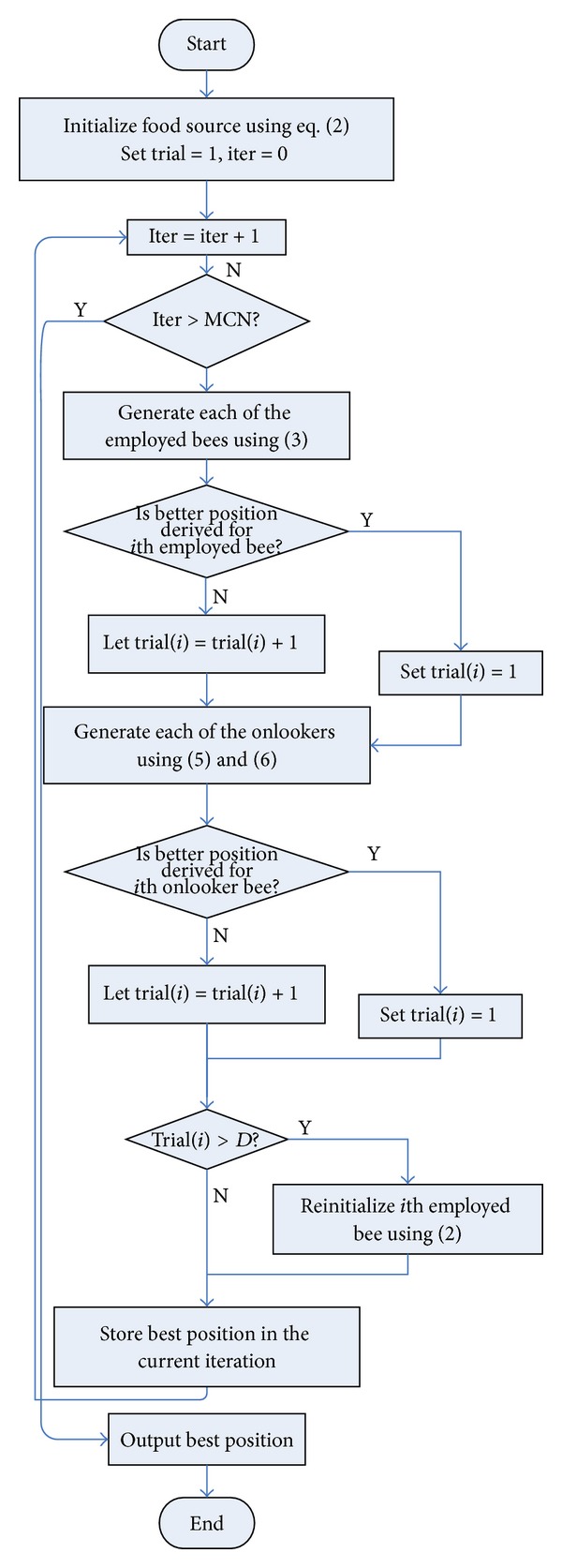
Flow chart of IF-ABC algorithm.

Figure 2 illustrates the flow chart of our IF-ABC algorithm. The pseudocode of IF-ABC for numerical optimization is given in Algorithm 1.

## 5. Experimental Results

In this section, besides the conventional ABC and IF-ABC, two state-of-the-art versions of ABC, named Gbest-ABC [41] and I-ABC [42], were tested on four ideal template matching cases as well as four practical cases. All the simulations were implemented in MATLAB R2010a and executed on an Intel Core 2 Duo CPU with 2 GB RAM running at 2.53 GHz under Windows XP. Every single type of experiment was repeated 500 times with different random seeds. User-specified parametric settings for these ABCs are listed in Table 1, where Limit denotes invalid trial time and SN stands for half of the swarm population. Satellite images involved in this paper originate from the Google Earth (visit http://www.google.com/earth/).

**Figure 3:**
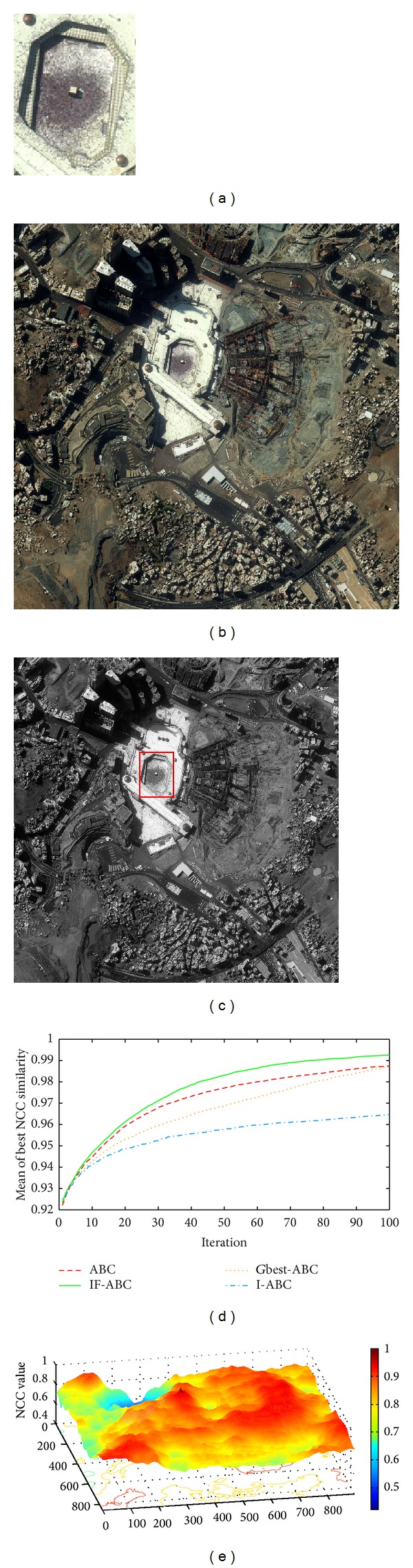
Ideal template matching case 1: satellite imagery of Holy City in Mecca. (a) Predefined template image (107 × 142); (b) source image (1000 × 1002); (c) ground matching truth; (d) comparative evolution curves of four ABC algorithms; (e) surface illustration of searching space.

**Figure 4:**
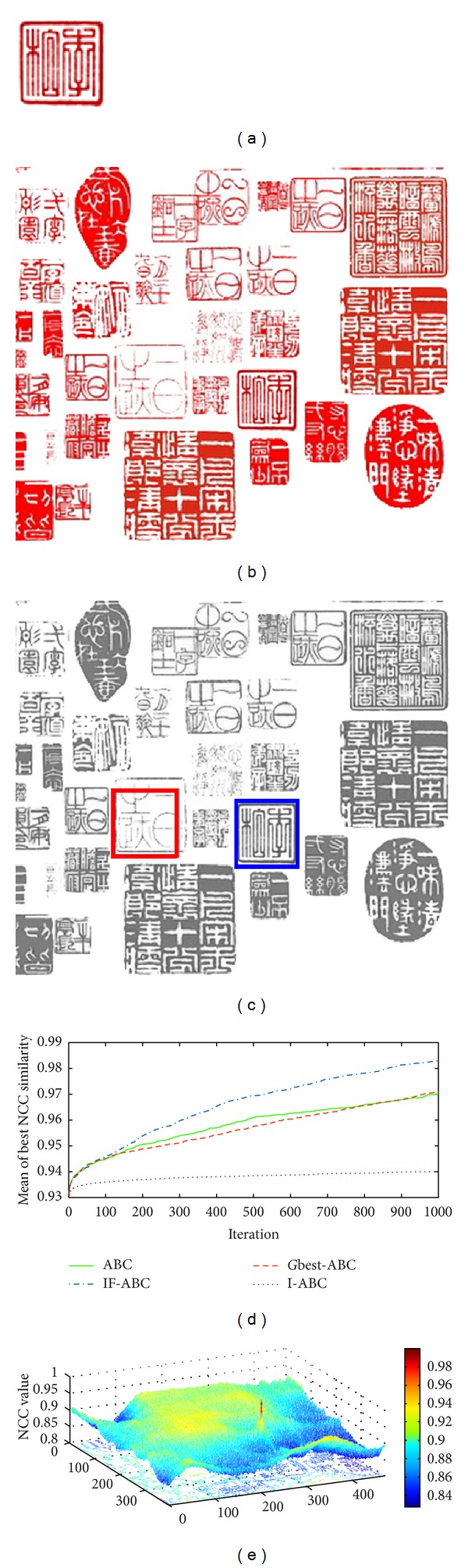
Ideal template matching case 2: traditional Chinese seal patterns. (a) Predefined template image (84 × 88); (b) source image (552 × 479); (c) ground matching truth (blue box) and most common false matching result (red box); (d) comparative evolution curves of four ABC algorithms; (e) surface illustration of searching space.

**Figure 5:**
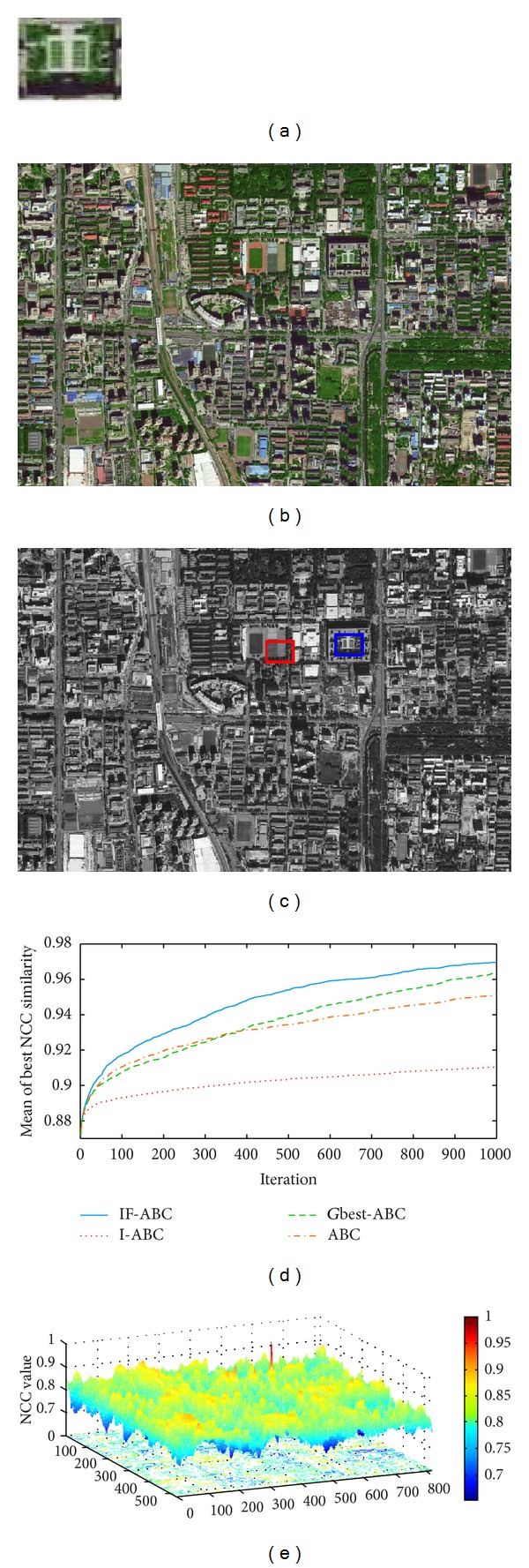
Ideal template matching case 3: satellite imagery of Beihang University campus. (a) Predefined template image (51 × 41); (b) source image (870 × 570); (c) ground matching truth (blue box) and most common false matching result (red box); (d) comparative evolution curves of four ABC algorithms; (e) surface illustration of searching space.

**Figure 6:**
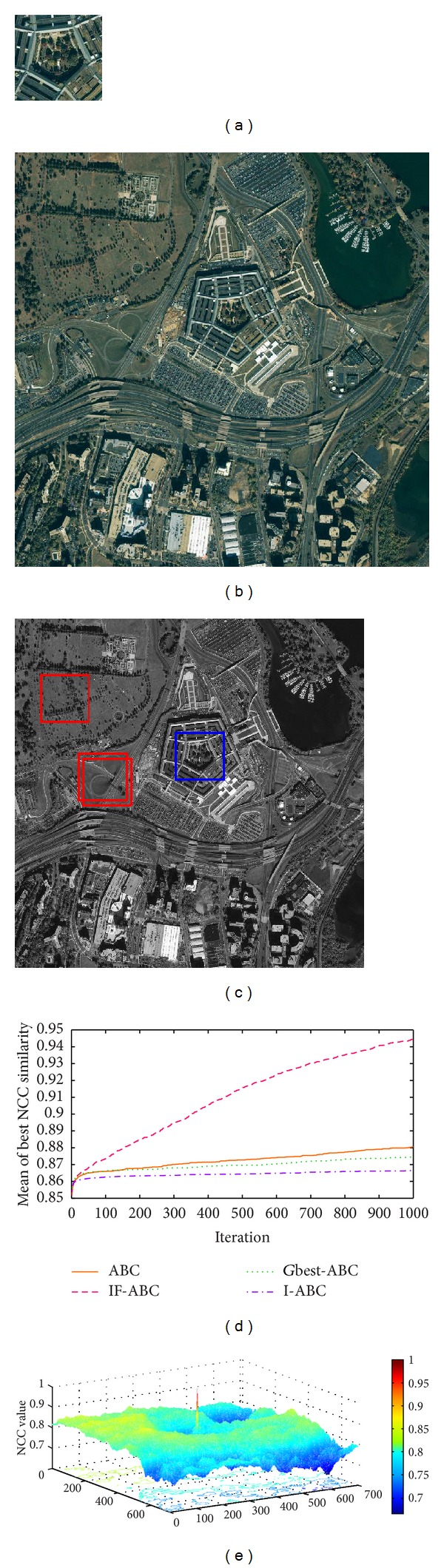
Ideal template matching case 4: satellite imagery of the Pentagon. (a) Predefined template image (116 × 114); (b) source image (820 × 820); (c) ground matching truth (blue box) and most common false matching results (red boxes); (d) comparative evolution curves of four ABC algorithms; (e) surface illustration of searching space.

Figures 3, 4, 5, and 6 illustrate the comparative simulation results concerning the four ideal cases; in each case the template is exactly part of the source image. Some typical local optimal matching locations are plotted (see the red boxes in Figures 3(c), 4(c), 5(c), and 6(c)), together with the global optimal matching locations (see the blue boxes). For the convenience of evaluation, two indexes that reflect the convergence performances (i.e., the mean and convergence rate C.R.) are listed in Table 2, where MCN stands for the predefined maximum cycle number. In order to show the necessity of applying evolutionary algorithms, we also made comparisons regarding the average time consumptions between IF-ABC algorithm and the classical full search algorithm in the four template matching cases.  Figure 11 demonstrates the comparative results, where each pair of comparison was repeated 50 times.

**Figure 7:**
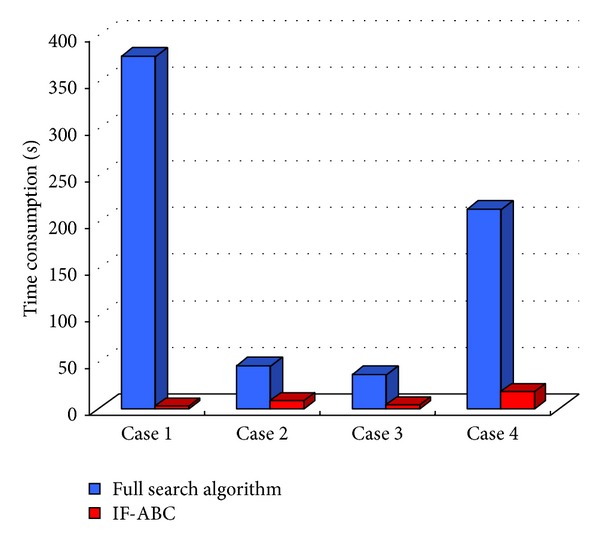
Comparisons of time consumptions between full search algorithm and IF-ABC in four ideal cases.

**Figure 8:**
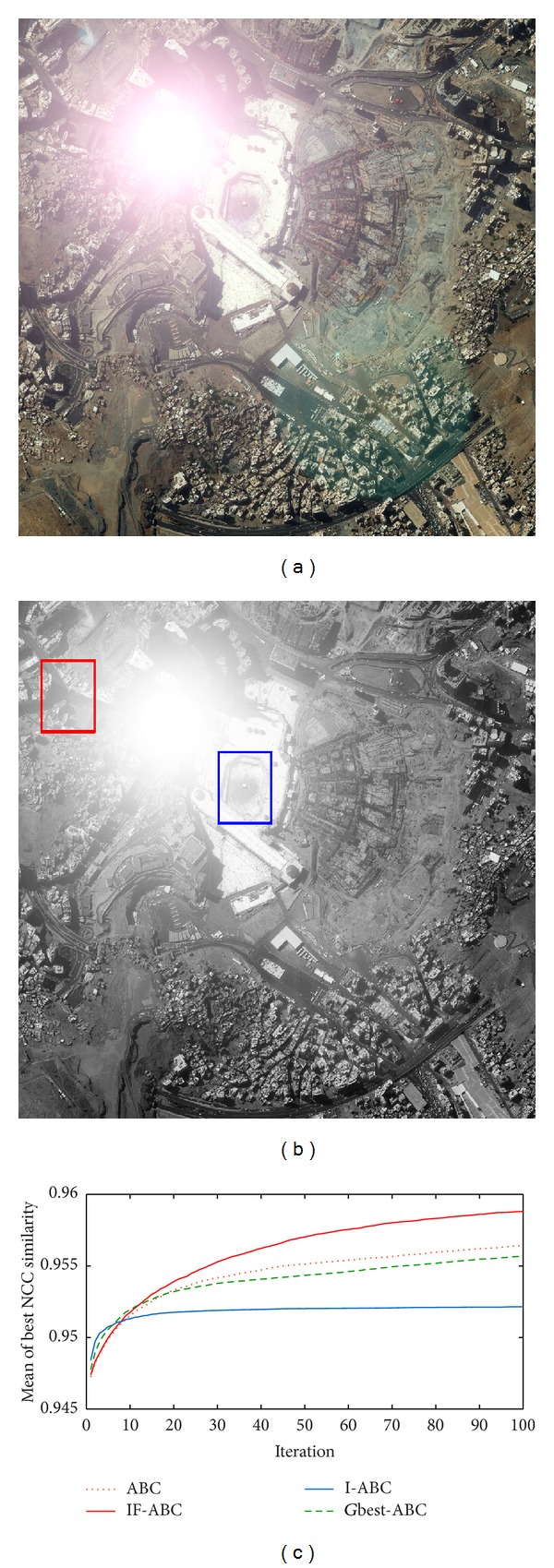
Practical template matching case 1. (a) Nonuniform illumination disturbed source image; (b) ground matching truth (blue box) and most common false matching result (red box); (c) comparative evolution curves of four ABC algorithms.

**Figure 9:**
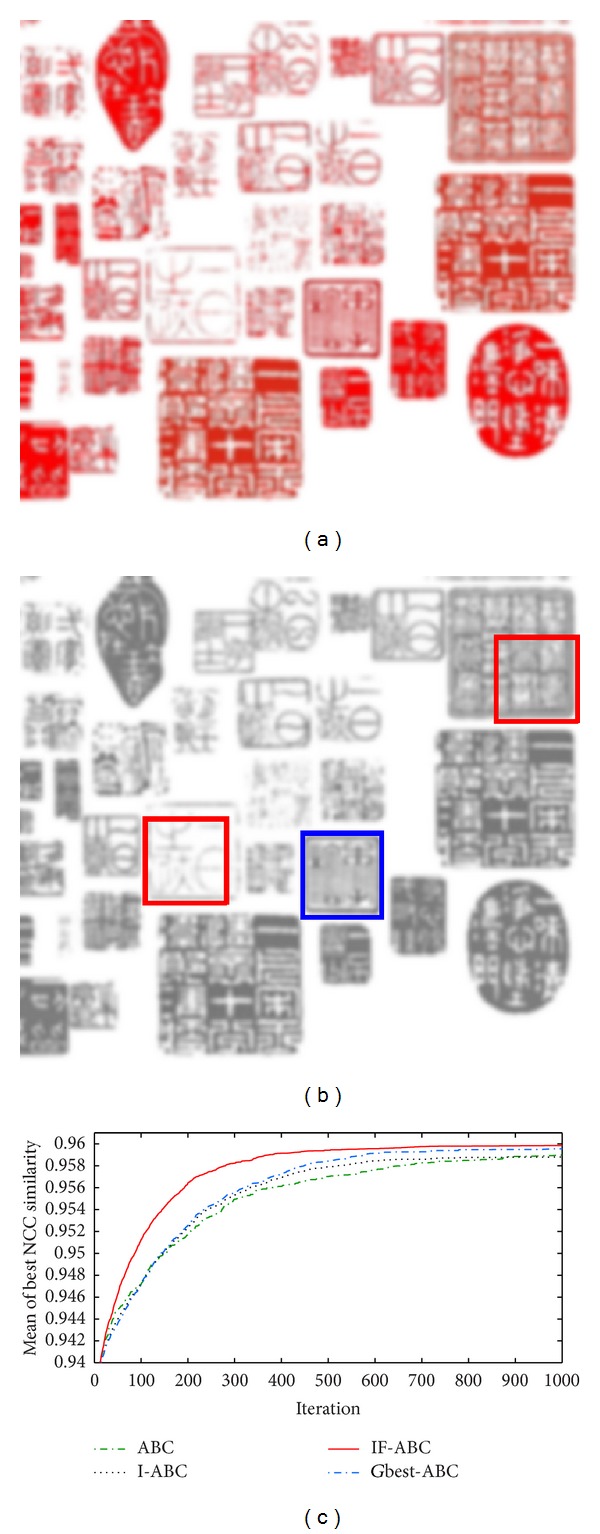
Practical template matching case 2. (a) Gaussian blurred source image; (b) ground matching truth (blue box) and most common false matching results (red boxes); (c) comparative evolution curves of four ABC algorithms.

**Figure 10:**
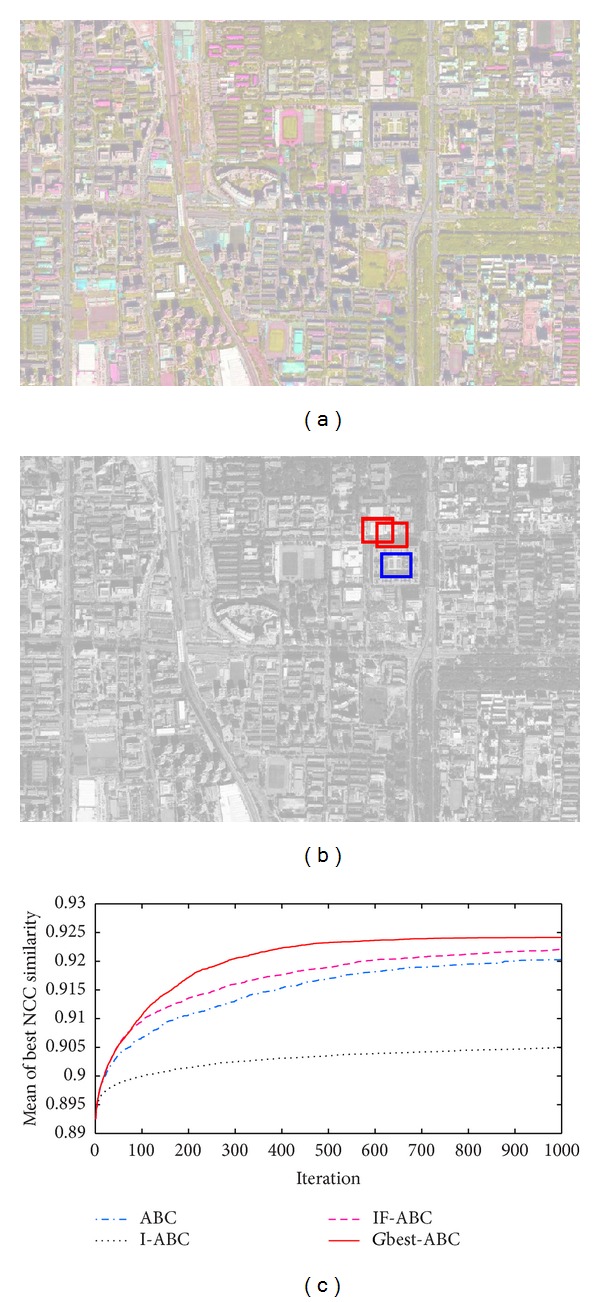
Practical template matching case 3. (a) Overexposed source image; (b) ground matching truth (blue box) and most common false matching results (red boxes); (c) comparative evolution curves of four ABC algorithms.

**Figure 11:**
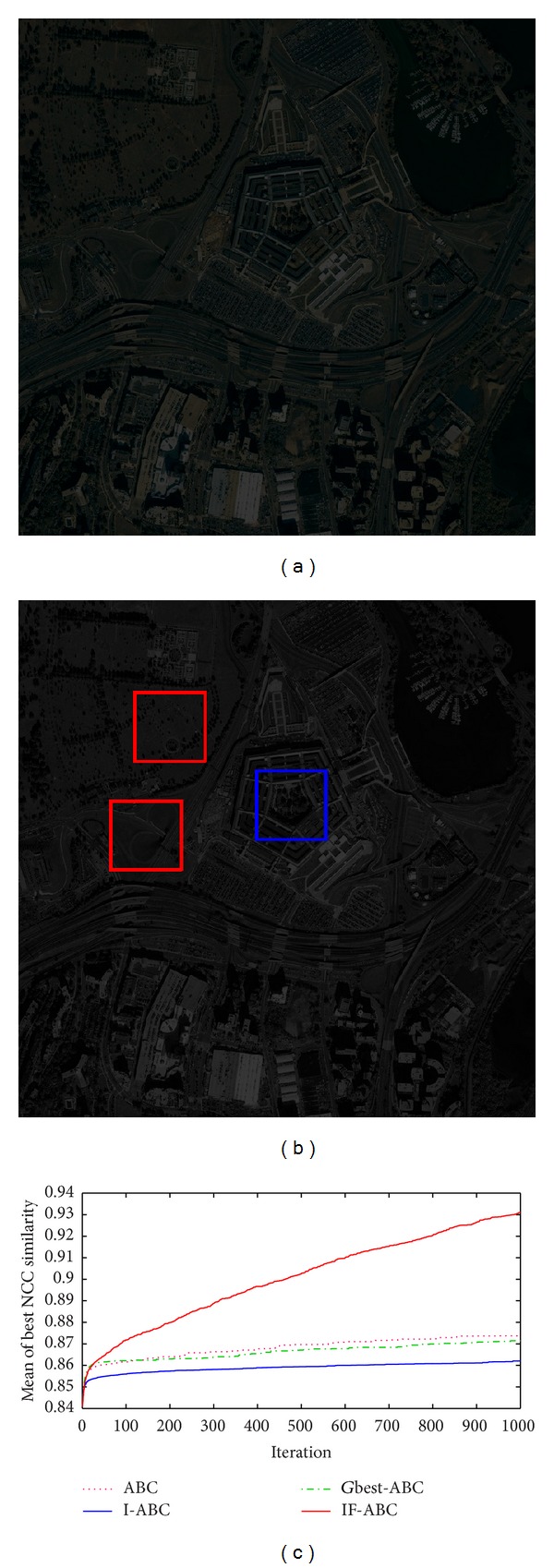
Practical template matching case 4. (a) Underexposed source image; (b) ground matching truth (blue box) and most common false matching results (red boxes); (c) comparative evolution curves of four ABC algorithms.

Similarly, another four cases of experiments with illumination disturbances considered were conducted. The results are shown in Figures 7, 8, 9, and 10, and the detailed comparisons are listed in Table 3.

Since template matching process does not take rotation or rescaling into consideration in our study, the two-dimensional domain is discrete (i.e., there exist a finite number of feasible solutions in total). Therefore, feasible solutions on a continuous domain need to be discretized so as to fit the objective function as in (1). As the objective function under consideration is a real-valued function of two variables, it is possible to plot the function surface using 3D graphics (as illustrated in Figures 3(e), 4(e), 5(e), and 6(e)).

## 6. Discussions

As shown in Table 2, the search process on the basis of IF-ABC is more efficient and robust than the other three algorithms. In detail, the convergence curves in Figures 3–6 suggest that the advantage of IF-ABC in case 1 is not as significant as it is in the rest of the three cases. It is also notable that the feasible solution surface in case 1 (see Figure 3(c)) is relatively smoother than the surfaces in the other three ideal cases (see Figures 4(c), 5(c), and 6(c)). In this particular case, classical methods are not inefficient. However, if the feasible solution surface is oscillatory or rugged, internal-feedback strategy will take effect. Take case 2 as an example, the overall surface is like a peak function (see Figure 4(c)), so it is more difficult to search for the global optimal location than case 1. The situations are quite similar in cases 3 and 4. In addition, although we observe from Table 2 that IF-ABC always possesses convergence rates far higher than those of the other three algorithms, the derivation of global optimum is not always guaranteed within the predefined iterations. Here comes the question: what is the advantage of IF-ABC in the case that a classical full search algorithm has been proposed nearly 40 years ago and always guarantees the derivation of a perfect matching result? Figure 7 gives a direct answer: the time consumption. For example, it takes IF-ABC an average of 3.6412 seconds to help find the optimal location (x*, y*) with NCC(x*, y*) = 1 in case 1, but it takes 378.9624 seconds for the full search algorithm. We conclude that it may be an epitome that accounts for the prosperity of developments in intelligent algorithms.

Experiments mentioned above are based on a default assumption that a source image does perfectly contain the predefined template image. However, such assumption is never satisfied in practical terms. Therefore, to testify the practical value of template matching (concerning the utilization of NCC model), four practical cases were added in our work. The original source images are blurred, overexposed, or underexposed in those practical cases, and the advantages of IF-ABC are still remarkable, expect for practical case 3 (see Figure 10). We still are not aware of what makes the situation different. But we did not intend to conceal this truth. It should be recognized that there is still room for improvement in IF-ABC.

IF-ABC is mainly highlighted by its ability to fight against premature convergence. On one hand, IF-ABC discards the well-known roulette selection procedure. On the other hand, it advocates to fully utilize the variables or indexes hiding in the convergence system, rather than to seek for some hybridization from the “outside world.” The authors believe that the roulette selection procedure does contribute a lot when optimizing some continuous unimodal objective functions. Nevertheless, when the objective function is discontinuous or multimodal, the roulette selection procedure will cause premature convergence because swarm diversity will be significantly reduced. In our point of view, convergence efficiency of the bees should be measured not by the corresponding objective function values, but by the fact whether they are better than the previous one. In this sense, it provides more possibilities for the so-called unqualified employed bees to be exploited locally by onlooker bees in the IF-ABC algorithm.

## 7. Conclusions and Future Work

The innovation of this work lies in the application of our previously proposed IF-ABC algorithm to solve the template matching problem. Experimental results clearly demonstrated the efficiency of IF-ABC in comparison with some state-of-the-art ABCs. Besides, it is also preliminarily confirmed that the NCC model works robustly when the source image can not 100% match the template image.

Our future work will focus on adopting this approach to some more complicated applications in the field of aeronautics and astronautics. Besides that, the efficiencies and robustness of different similarity measurement models (including the NCC model) under nonuniform illumination conditions will be theoretically evaluated.

## Figures and Tables

**Algorithm 1:**
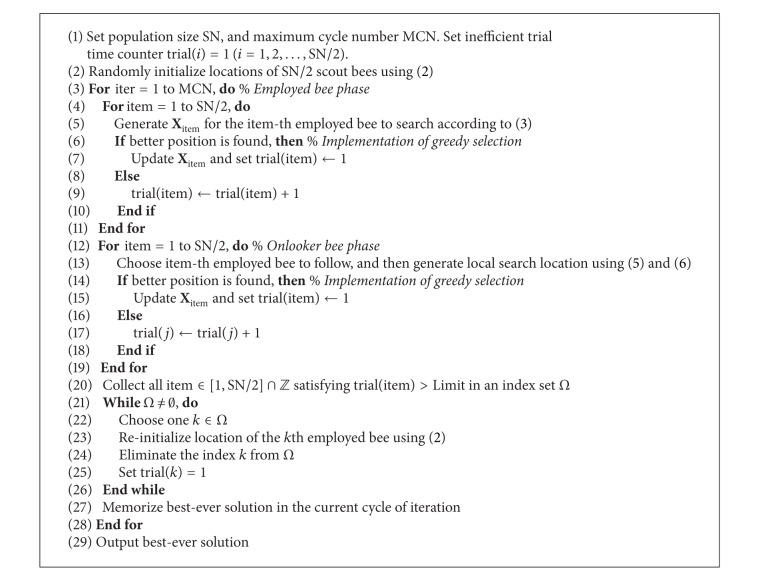
titleworktilte

**Table 1:** Parameter settings for four ABCs.

ABC	I-ABC	Gbest-ABC	IF-ABC
SN = 20	SN = 20	SN = 20	SN = 20
Limit = 10	Limit = 10	Limit = 10	Limit = 10
	ap = fitness(1)|_iter=1_	*C* = 2	*α* = 0.1

**Table 2:** Comparative convergence performances of four ABCs.

Case number	MCN	ABC		I-ABC		Gbest-ABC		IF-ABC	
		Mean	C.R.	Mean	C.R.	Mean	C.R.	Mean	C.R.
1	100	0.9874	39.60%	0.9647	1.20%	0.9870	58.80%	**0.9927**	**79.80%**
2	1000	0.9701	25.40%	0.9400	0.40%	0.9709	36.40%	**0.9829**	**57.80%**
3	1000	0.9517	22.00%	0.9103	1.80%	0.9637	41.40%	**0.9698**	**50.60%**
4	1000	0.8806	3.00%	0.8664	0.20%	0.8746	1.80%	**0.9445**	**44.20%**

*Those bold values denote the best value (mean or C. R.) in every single line.

**Table 3:** Comparative convergence performances of four ABC relevant algorithms.

Case number	MCN	ABC	I-ABC	Gbest-ABC	IF-ABC
		Mean	Mean	Mean	Mean
1	100	0.9564	0.9521	0.9557	**0.9588**
2	1000	0.9588	0.9588	0.9596	**0.9598**
3	1000	0.9197	0.9055	**0.9241**	0.9221
4	1000	0.8746	0.8613	0.8718	**0.9310**

*Those bold values denote the best mean value in every single line.
